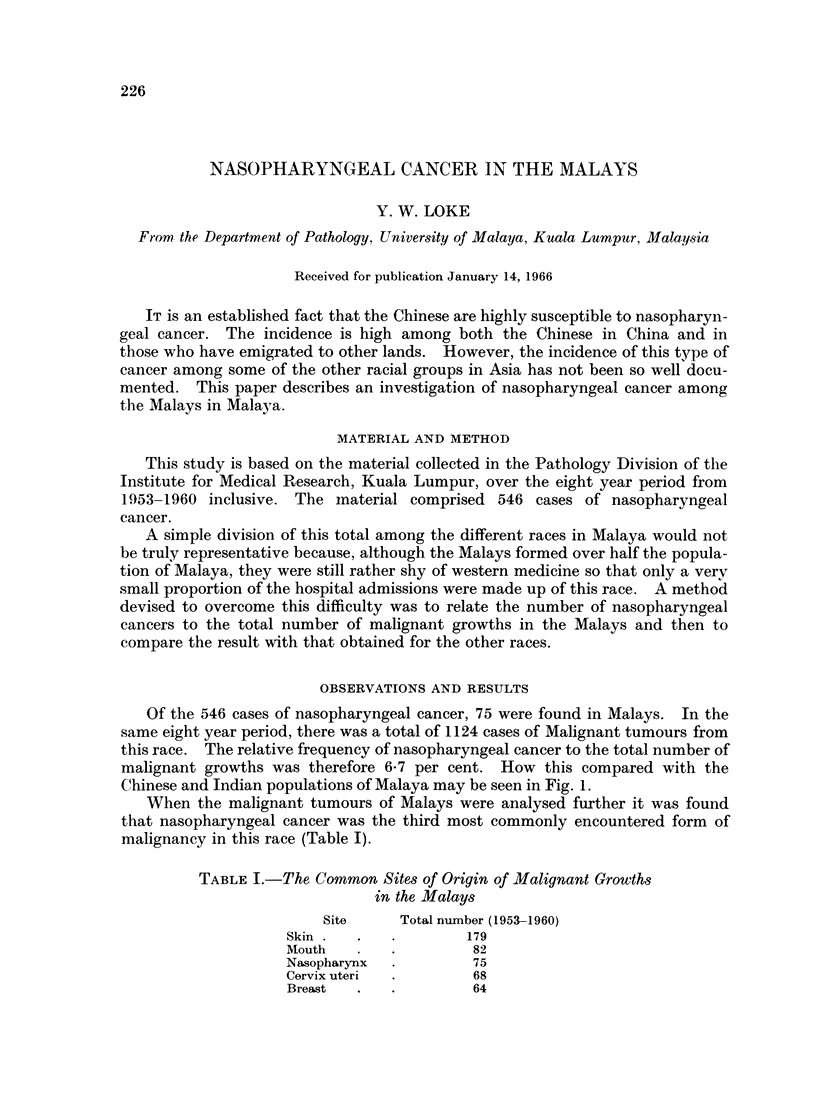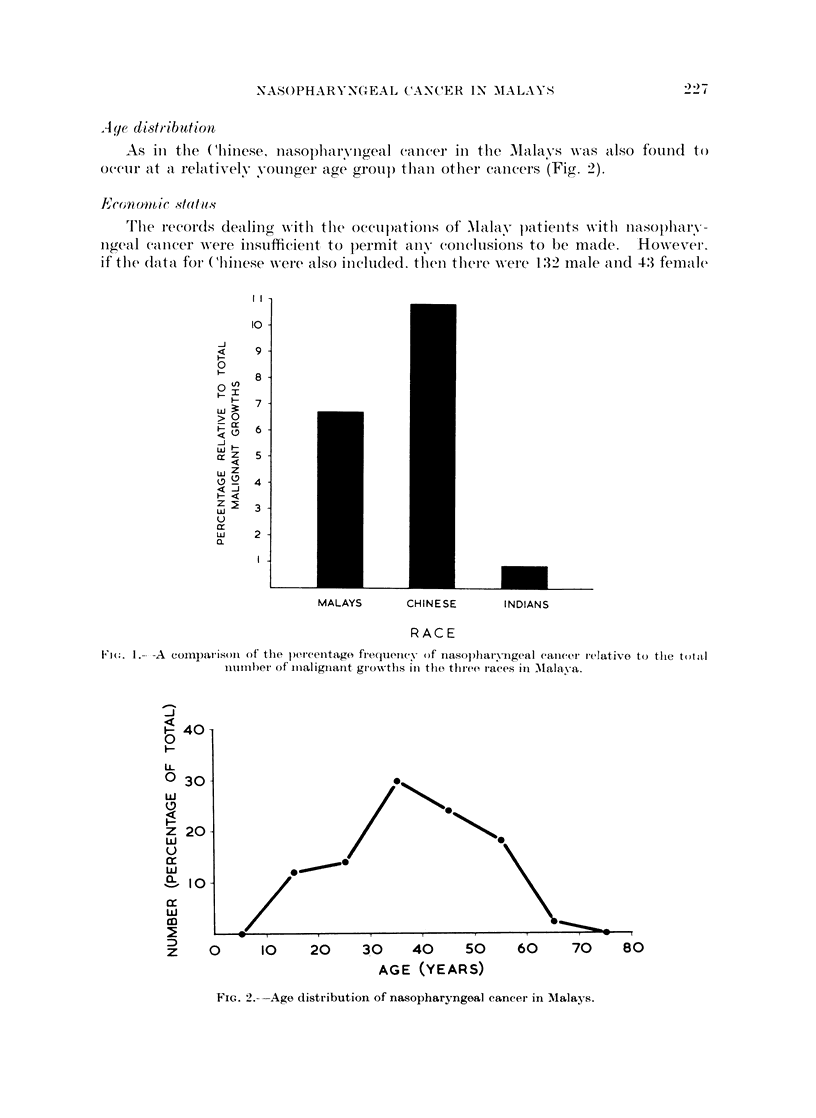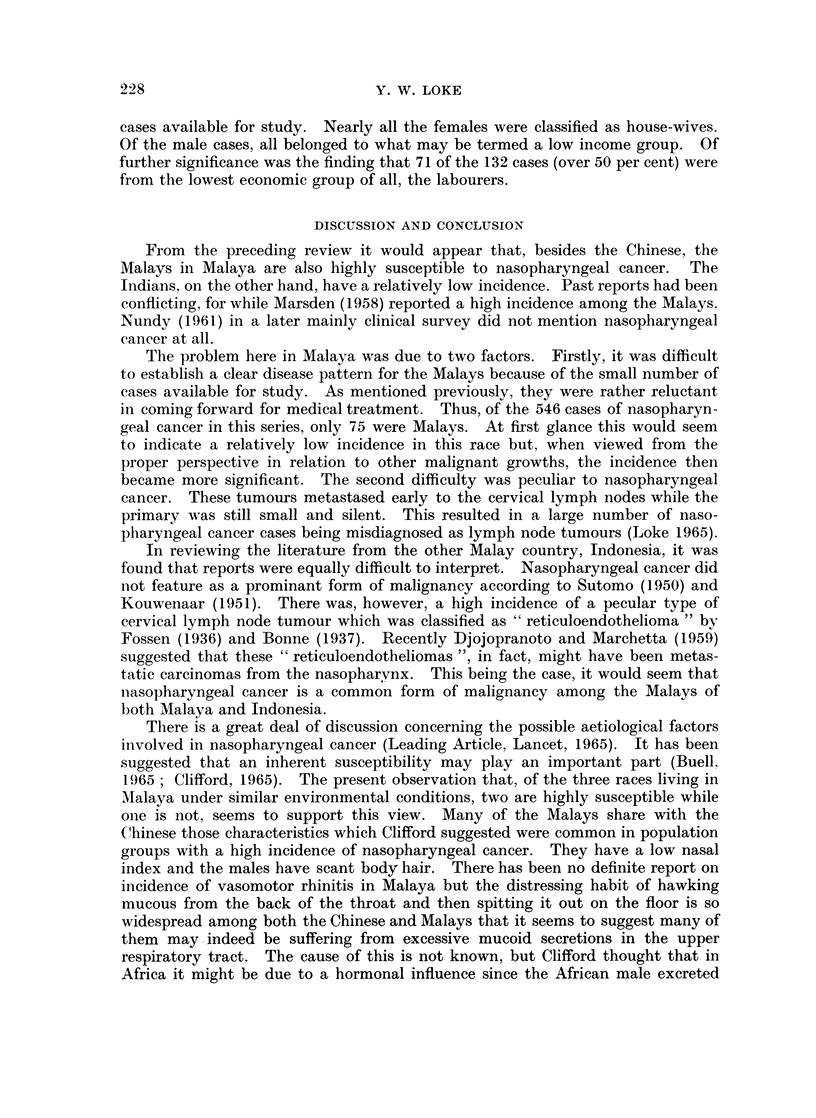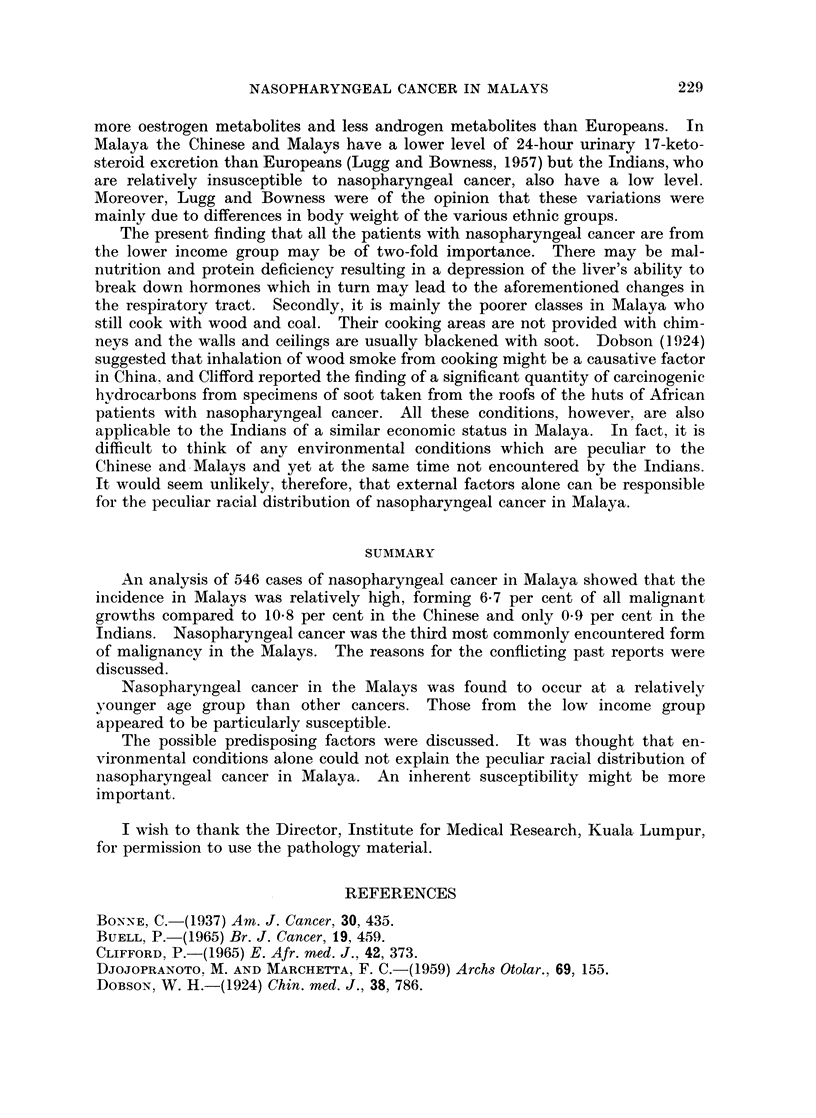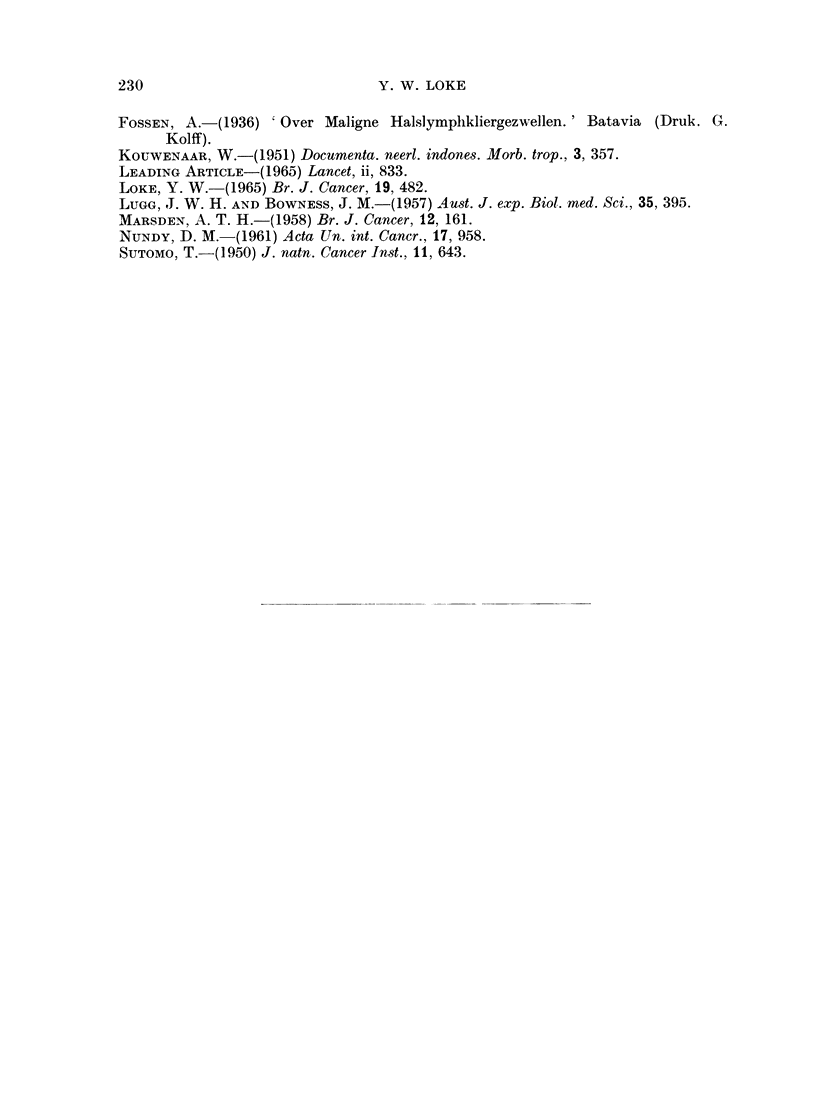# Nasopharyngeal cancer in the Malays.

**DOI:** 10.1038/bjc.1966.27

**Published:** 1966-06

**Authors:** Y. W. Loke


### NASOPHARYNGEAL CANCER IN THE MALAYS

Y. W. LOKE

*From the Department of Pathology, University of Malaya, Kuala Lumpur, Malaysia*

Received for publication January 14, 1966

It is an established fact that the Chinese are highly susceptible to nasopharyngeal cancer. The incidence is high among both the Chinese in China and in those who have emigrated to other lands. However, the incidence of this type of cancer among some of the other racial groups in Asia has not been so well documented. This paper describes an investigation of nasopharyngeal cancer among the Malays in Malaya.

### MATERIAL AND METHOD

This study is based on the material collected in the Pathology Division of the Institute for Medical Research, Kuala Lumpur, over the eight year period from 1953–1960 inclusive. The material comprised 546 cases of nasopharyngeal cancer.

A simple division of this total among the different races in Malaya would not be truly representative because, although the Malays formed over half the population of Malaya, they were still rather shy of western medicine so that only a very small proportion of the hospital admissions were made up of this race. A method devised to overcome this difficulty was to relate the number of nasopharyngeal cancers to the total number of malignant growths in the Malays and then to compare the result with that obtained for the other races.

### OBSERVATIONS AND RESULTS

Of the 546 cases of nasopharyngeal cancer, 75 were found in Malays. In the same eight year period, there was a total of 1124 cases of Malignant tumours from this race. The relative frequency of nasopharyngeal cancer to the total number of malignant growths was therefore 6·7 per cent. How this compared with the Chinese and Indian populations of Malaya may be seen in Fig. 1.

When the malignant tumours of Malays were analysed further it was found that nasopharyngeal cancer was the third most commonly encountered form of malignancy in this race (Table I).

TABLE I.—*The Common Sites of Origin of Malignant Growths in the Malays*

| Site | Total number (1953–1960) |
|---|---|
| Skin | 179 |
| Mouth | 82 |
| Nasopharynx | 75 |
| Cervix uteri | 68 |
| Breast | 64 |

*Age distribution*

As in the Chinese, nasopharyngeal cancer in the Malays was also found to occur at a relatively younger age group than other cancers (Fig. 2).

*Economic status*

The records dealing with the occupations of Malay patients with nasopharyngeal cancer were insufficient to permit any conclusions to be made. However, if the data for Chinese were also included, then there were 132 male and 43 female

FIG. 1.—A comparison of the percentage frequency of nasopharyngeal cancer relative to the total number of malignant growths in the three races in Malaya.

FIG. 2.—Age distribution of nasopharyngeal cancer in Malays.

cases available for study. Nearly all the females were classified as house-wives. Of the male cases, all belonged to what may be termed a low income group. Of further significance was the finding that 71 of the 132 cases (over 50 per cent) were from the lowest economic group of all, the labourers.

### DISCUSSION AND CONCLUSION

From the preceding review it would appear that, besides the Chinese, the Malays in Malaya are also highly susceptible to nasopharyngeal cancer. The Indians, on the other hand, have a relatively low incidence. Past reports had been conflicting, for while Marsden (1958) reported a high incidence among the Malays, Nundy (1961) in a later mainly clinical survey did not mention nasopharyngeal cancer at all.

The problem here in Malaya was due to two factors. Firstly, it was difficult to establish a clear disease pattern for the Malays because of the small number of cases available for study. As mentioned previously, they were rather reluctant in coming forward for medical treatment. Thus, of the 546 cases of nasopharyngeal cancer in this series, only 75 were Malays. At first glance this would seem to indicate a relatively low incidence in this race but, when viewed from the proper perspective in relation to other malignant growths, the incidence then became more significant. The second difficulty was peculiar to nasopharyngeal cancer. These tumours metastased early to the cervical lymph nodes while the primary was still small and silent. This resulted in a large number of nasopharyngeal cancer cases being misdiagnosed as lymph node tumours (Loke 1965).

In reviewing the literature from the other Malay country, Indonesia, it was found that reports were equally difficult to interpret. Nasopharyngeal cancer did not feature as a prominant form of malignancy according to Sutomo (1950) and Kouwenaar (1951). There was, however, a high incidence of a pecular type of cervical lymph node tumour which was classified as " reticuloendothelioma " by Fossen (1936) and Bonne (1937). Recently Djojopranoto and Marchetta (1959) suggested that these " reticuloendotheliomas ", in fact, might have been metastatic carcinomas from the nasopharynx. This being the case, it would seem that nasopharyngeal cancer is a common form of malignancy among the Malays of both Malaya and Indonesia.

There is a great deal of discussion concerning the possible aetiological factors involved in nasopharyngeal cancer (Leading Article, Lancet, 1965). It has been suggested that an inherent susceptibility may play an important part (Buell, 1965 ; Clifford, 1965). The present observation that, of the three races living in Malaya under similar environmental conditions, two are highly susceptible while one is not, seems to support this view. Many of the Malays share with the Chinese those characteristics which Clifford suggested were common in population groups with a high incidence of nasopharyngeal cancer. They have a low nasal index and the males have scant body hair. There has been no definite report on incidence of vasomotor rhinitis in Malaya but the distressing habit of hawking mucous from the back of the throat and then spitting it out on the floor is so widespread among both the Chinese and Malays that it seems to suggest many of them may indeed be suffering from excessive mucoid secretions in the upper respiratory tract. The cause of this is not known, but Clifford thought that in Africa it might be due to a hormonal influence since the African male excreted more oestrogen metabolites and less androgen metabolites than Europeans. In Malaya the Chinese and Malays have a lower level of 24-hour urinary 17-keto-steroid excretion than Europeans (Lugg and Bowness, 1957) but the Indians, who are relatively insusceptible to nasopharyngeal cancer, also have a low level. Moreover, Lugg and Bowness were of the opinion that these variations were mainly due to differences in body weight of the various ethnic groups.

The present finding that all the patients with nasopharyngeal cancer are from the lower income group may be of two-fold importance. There may be malnutrition and protein deficiency resulting in a depression of the liver's ability to break down hormones which in turn may lead to the aforementioned changes in the respiratory tract. Secondly, it is mainly the poorer classes in Malaya who still cook with wood and coal. Their cooking areas are not provided with chimneys and the walls and ceilings are usually blackened with soot. Dobson (1924) suggested that inhalation of wood smoke from cooking might be a causative factor in China, and Clifford reported the finding of a significant quantity of carcinogenic hydrocarbons from specimens of soot taken from the roofs of the huts of African patients with nasopharyngeal cancer. All these conditions, however, are also applicable to the Indians of a similar economic status in Malaya. In fact, it is difficult to think of any environmental conditions which are peculiar to the Chinese and Malays and yet at the same time not encountered by the Indians. It would seem unlikely, therefore, that external factors alone can be responsible for the peculiar racial distribution of nasopharyngeal cancer in Malaya.

### SUMMARY

An analysis of 546 cases of nasopharyngeal cancer in Malaya showed that the incidence in Malays was relatively high, forming 6·7 per cent of all malignant growths compared to 10·8 per cent in the Chinese and only 0·9 per cent in the Indians. Nasopharyngeal cancer was the third most commonly encountered form of malignancy in the Malays. The reasons for the conflicting past reports were discussed.

Nasopharyngeal cancer in the Malays was found to occur at a relatively younger age group than other cancers. Those from the low income group appeared to be particularly susceptible.

The possible predisposing factors were discussed. It was thought that environmental conditions alone could not explain the peculiar racial distribution of nasopharyngeal cancer in Malaya. An inherent susceptibility might be more important.

I wish to thank the Director, Institute for Medical Research, Kuala Lumpur, for permission to use the pathology material.

### REFERENCES

Bonne, C.—(1937) *Am. J. Cancer*, **30**, 435.
Buell, P.—(1965) *Br. J. Cancer*, **19**, 459.
Clifford, P.—(1965) *E. Afr. med. J.*, **42**, 373.
Djojopranoto, M. and Marchetta, F. C.—(1959) *Archs Otolar.*, **69**, 155.
Dobson, W. H.—(1924) *Chin. med. J.*, **38**, 786.

FOSSEN, A.—(1936) 'Over Maligne Halslymphkliergezwellen.' Batavia (Druk. G. Kolff).
KOUWENAAR, W.—(1951) *Documenta. neerl. indones. Morb. trop.*, **3**, 357.
LEADING ARTICLE—(1965) *Lancet*, ii, 833.
LOKE, Y. W.—(1965) *Br. J. Cancer*, **19**, 482.
LUGG, J. W. H. AND BOWNESS, J. M.—(1957) *Aust. J. exp. Biol. med. Sci.*, **35**, 395.
MARSDEN, A. T. H.—(1958) *Br. J. Cancer*, **12**, 161.
NUNDY, D. M.—(1961) *Acta Un. int. Cancr.*, **17**, 958.
SUTOMO, T.—(1950) *J. natn. Cancer Inst.*, **11**, 643.